# A Pharmacologic Approach to Acquired Cystic Fibrosis Transmembrane Conductance Regulator Dysfunction in Smoking Related Lung Disease

**DOI:** 10.1371/journal.pone.0039809

**Published:** 2012-06-29

**Authors:** Peter A. Sloane, Suresh Shastry, Andrew Wilhelm, Clifford Courville, Li Ping Tang, Kyle Backer, Elina Levin, S. Vamsee Raju, Yao Li, Marina Mazur, Suzanne Byan-Parker, William Grizzle, Eric J. Sorscher, Mark T. Dransfield, Steven M. Rowe

**Affiliations:** 1 Department of Medicine, University of Alabama at Birmingham, Birmingham, Alabama, United States of America; 2 Department of Pediatrics, University of Alabama at Birmingham, Birmingham, Alabama, United States of America; 3 Department of Physiology and Biophysics, University of Alabama at Birmingham, Birmingham, Alabama, United States of America; 4 Department of Pathology, University of Alabama at Birmingham, Birmingham, Alabama, United States of America; 5 UAB Lung Health Center, University of Alabama at Birmingham, Birmingham, Alabama, United States of America; 6 Cystic Fibrosis Research Center, University of Alabama at Birmingham, Birmingham, Alabama, United States of America; Abramson Research Center, United States of America

## Abstract

**Background:**

Mucus stasis in chronic obstructive pulmonary disease (COPD) is a significant contributor to morbidity and mortality. Potentiators of cystic fibrosis transmembrane conductance regulator (CFTR) activity pharmacologically enhance CFTR function; ivacaftor is one such agent approved to treat CF patients with the G551D-CFTR gating mutation. CFTR potentiators may also be useful for other diseases of mucus stasis, including COPD.

**Methods and Findings:**

In primary human bronchial epithelial cells, exposure to cigarette smoke extract diminished CFTR-mediated anion transport (65.8±0.2% of control, P<0.005) and mucociliary transport (0.17±0.05 µm/sec vs. 2.4±0.47 µm/sec control, P<0.05) by reducing airway surface liquid depth (7.3±0.6 µm vs. 13.0±0.6 µm control, P<0.005) and augmenting mucus expression (by 64%, P<0.05) without altering transepithelial resistance. Smokers with or without COPD had reduced CFTR activity measured by nasal potential difference compared to age-matched non-smokers (−6.3±1.4 and −8.0±2.0 mV, respectively vs. −15.2±2.7 mV control, each P<0.005, n = 12–14/group); this CFTR decrement was associated with symptoms of chronic bronchitis as measured by the Breathlessness Cough and Sputum Score (r = 0.30, P<0.05) despite controlling for smoking (r = 0.31, P<0.05). Ivacaftor activated CFTR-dependent chloride transport in non-CF epithelia and ameliorated the functional CFTR defect induced by smoke to 185±36% of non-CF control (P<0.05), thereby increasing airway surface liquid (from 7.3±0.6 µm to 10.1±0.4 µm, P<0.005) and mucociliary transport (from 0.27±0.11 µm/s to 2.7±0.28 µm/s, P<0.005).

**Conclusions:**

Cigarette smoking reduces CFTR activity and is causally related to reduced mucus transport in smokers due to inhibition of CFTR dependent fluid transport. These effects are reversible by the CFTR potentiator ivacaftor, representing a potential therapeutic strategy to augment mucociliary clearance in patients with smoking related lung disease.

## Introduction

Cystic fibrosis transmembrane conductance regulator (CFTR) is an epithelial anion channel expressed predominantly in exocrine tissues. Mutations in the gene encoding CFTR are the proximate cause of CF [1]. In the lung, loss of CFTR function results in airway surface liquid (ASL) depletion, reduced mucociliary clearance (MCC), chronic bacterial infection, and excess inflammation [2]. Over time, pulmonary obstruction due to inspisated respiratory secretions and bronchiectasis ensues, resulting in respiratory failure.

Individuals with smoking-induced lung disease, and in particular those with chronic obstructive pulmonary disease (COPD) associated with chronic bronchitis [3], [4], exhibit pathologic features similar to CF, including mucus stasis and accumulation [4], [5], [6]. While some COPD patients develop bronchiectasis [7], most exhibit chronic bacterial colonization and persistent neutrophilic airway inflammation, pathophysiologic processes reminiscent of CF lung disease [8], [9]. Mucus accumulation in COPD is independently associated with lung function decline, exacerbation frequency, and early mortality [10], [11], [12]. At present, no therapies definitively address mucus accumulation in the COPD lung.

Emerging data suggest exposure to cigarette smoke causes CFTR dysfunction in the upper airways. Cigarette smoke inhibits chloride transport in cultured bronchial epithelial cells [13], [14], a finding supported *in vivo* by reduced CFTR-dependent nasal potential difference (NPD) in the upper airways of healthy smokers without CFTR mutations [13]. The development of efficacious modulators of CFTR anion transport has raised the possibility that pharmacologic enhancement of CFTR activity may have important clinical significance, even among individuals without congenital *CFTR* mutations. The novel CFTR potentiator ivacaftor (Kalydeco™, VX-770) was recently approved for use in CF patients with the G551D-CFTR gating mutation based on marked improvements in multiple clinical endpoints in phase 2 and 3 trials [15], [16]. Ivacaftor robustly enhances anion secretion by potentiating cAMP mediated CFTR channel gating [17] leading to airway fluid secretion, and points to novel treatment strategies for common diseases of mucus stasis, including COPD caused by cigarette smoking [18]. In these studies we tested the hypothesis that cigarette exposure has deleterious effects on mucociliary transport by causing an imbalance of CFTR mediated fluid secretion and enhanced mucus expression. Complementary studies in smokers with and without COPD suggested CFTR dysfunction is present, and thus may represent a viable therapeutic target. Our *in vitro* studies establish that the functional CFTR deficiency caused by smoke exposure can be ameliorated by addition of ivacaftor, thereby augmenting airway surface liquid depth and mucociliary transport. These findings suggest that CFTR potentiators may offer a new treatment paradigm for COPD associated with chronic bronchitis.

## Methods

### Procurement and Growth of Primary Airway Epithelial Cells

Use of human cells and tissues was approved by the UAB Institutional Review Board. Primary human bronchial epithelial cells were derived from lung explants after written informed consent was obtained from CF and non-CF subjects with confirmed CFTR genetics by methods described previously [17], [19]. Briefly, tissues were debrided immediately after surgical resection, washed twice in Minimum Essential Media with 0.5 mg/ml DTT (Sigma-Aldrich, St. Louis, MO) and 25 U/ml DNAse I (Roche, Basel, Switzerland), and then placed in dissociation media containing MEM, 2.5 U/ml DNAse I, 100 µg/ml ceftazidime, 80 µg/ml tobramycin, 1.25 µg/ml amphotericin B, and 4.4 U/ml pronase (Sigma-Aldrich) for 24–36 h at 4°C. Loosened airway epithelial cells were then expanded in growth media containing BEGM (LONZA, Basel, Switzerland) supplemented with an additional 10 nM all trans-retinoic acid (Sigma-Aldrich) that was exchanged every 24 h. Following expansion, first or second passage cells were seeded on permeable supports for studies.

Once 80–90% confluent, cells were seeded on Snapwell 1.13 cm^2^ permeable supports (1×10^6^ cells/filter; Bayer, Pittsburgh, PN) or Costar 0.4 µm permeable supports (5×10^5^ cells/filter; Bethesda, MD) after coating with NIH 3T3 fibroblast conditioned media, and grown in differentiating media containing DMEM/F12 (Invitrogen, Carlsbad, California), 2% Ultroser-G (Pall, New York, NY), 2% Fetal Clone II (Hyclone, Logan, UT), 2.5 µg/ml Insulin (Sigma-Aldrich), 0.25% bovine brain extract (LONZA), 20 nM hydrocortisone (Sigma-Aldrich), 500 nM Triodothyronine (Sigma-Aldrich), 2.5 µg/ml transferrin (Invitrogen), 250 nM ethanolamine (Sigma-Aldrich), 1.5 µM epinephrine (Sigma-Aldrich), 250 nM phosphoetheanolamine (Sigma-Aldrich), and 10 nM all trans-retinoic acid until terminally differentiated, as previously described [17], [19].

### Voltage Clamp Studies in Ussing Chambers

Short-circuit current (Isc) was measured under voltage clamp conditions using MC8 voltage clamps and P2300 Ussing chambers (Physiologic Instruments, San Diego, CA) as previously described [19]. Monolayers were initially bathed on both sides with identical Ringer’s solutions containing (in mM) 115 NaCl, 25 NaHCO_3_, 2.4 KH_2_PO_4_, 1.24 K_2_HPO_4_, 1.2 CaCl_2_, 1.2 MgCl_2_, 10 D-glucose (pH 7.4). Bath solutions were vigorously stirred and gassed with 95%O_2_∶5% CO_2_. Short-circuit current measurements were obtained using an epithelial voltage clamp (Physiologic Instruments). A one-second three-mV pulse was imposed every 10 seconds to monitor resistance calculated using Ohm’s law. Where indicated, the mucosal bathing solution was changed to a low Cl^−^ solution containing 1.2 NaCl and 115 Na^+^ gluconate, and all other components as above. Amiloride (100 µM) was added to block residual Na^+^ current, followed by the agonists forskolin, ivacaftor, and ATP as indicated (minimum five-min observation at each concentration). CFTR_Inh_-172 (10 µM) was added to the mucosal bathing solution at the end of experiments to block CFTR-dependent Isc. All chambers were maintained at 37°C, and agonist stimulation was initiated within 15 min of placement into the chambers.

### CFTR Western Blotting

Cells were washed with ice-cold 1× PBS twice, then lysed with ice-cold RIPA buffer (150 mM NaCl, 1% Nonidet P-40, 0.5% sodium deoxycholate, 0.1% SDS, 50 mM Tris·HCl) containing protease inhibitors (Thermoscientific, Waltham, MA). Lysates were centrifuged at 14,000 g for 15 min at 4°C. Equal amounts of protein were electrophoresed on SDS-PAGE gels (Invitrogen, Carlsbad, CA) then transferred to nitrocellulose membranes (BioRad Laboratories, Hercules, CA). Blots were blocked in 1× PBS containing 5% (w/v) milk powder and 0.1% Tween-20, then incubated with 1∶500 diluted anti-CFTR antibody (Millipore Corporation, Billerica, MA) overnight at 4°C, washed, followed by secondary antibody (Dako, Carpinteria, CA) conjugated with horseradish peroxidase 1∶3000 for 1 h at room temperature. Chemiluminescence was induced with high-sensitivity Immobilon Western substrate (Millipore, Billerica, MA). The membranes were exposed using CemiDoc XRS HQ (Bio-Rad, Hercules, CA, USA) for different periods (up to 2 min) and calibrated in the linear range for a standard set of diluted samples [20].

### Cell-surface CFTR Biotinylation Studies

All cell surface glycoproteins were biotinylated similar to that previously described [19]. Biotinylated protein was immunoprecipitated using NeutrAvidin beads, separated by SDS-PAGE gel and detected by Western blot. CFTR was detected with anti-CFTR NBD1 10B6.2 polyclonal antibody followed by enhanced chemiluminescence (ECL; Pierce Biotechnology, Inc., Rockford, IL, USA).

### Real-Time Polymerase Chain Reaction (RT-PCR)

A TaqMan One-Step RT-PCR protocol (Applied Biosystems, Foster City, CA) was used to quantify CFTR mRNA transcripts using Assays on Demand Gene Expression Products, coupled with the ABI Prism 7500 sequence detection system (Applied Biosystems) as previously described for both nasal curettage and primary airway cell specimens [21], [22]. Briefly, total RNA was isolated using the Qiagen RNeasy mini kit according to manufacturer’s instructions. To prevent possible DNA contamination, samples were pretreated with RNase-free DNase (Qiagen, Valencia, CA). Sequence specific primers and probes for human *CFTR* and 18S rRNA were purchased from Assays on Demand (Applied Biosystems); Assay ID for *CFTR*: Hs00357011_m1. The probe extends across the exon 21/22 boundary of the human *CFTR* sequence. TaqMan One Step PCR Master Mix Reagents Kit (Applied Biosystems) was used for reverse transcription and PCR. The reaction volume was 25 µl and contained 12.5 µl of 2× Master Mix without uracil-N-glycosylase, 0.625 µl of 40× MultiScribe and RNase Inhibitor Mix, 1.25 µl of 20× target primer and probe, 5.625 µl of nuclease-free water (Ambion, Austin, TX), and 5 µl of RNA sample. Reaction plates were covered with an optical cap and centrifuged briefly to remove bubbles. Thermocycler conditions were as follows: Stage 1: 48°C for 30 min; Stage 2: 95°C for 10 min; Stage 3: 95°C for 15 sec, repeat 40 cycles, 60°C for 1 min. All experiments were run in triplicate for verification. The relative quantification of transcript levels (CFTR compared with endogenous 18S rRNA) was performed using the standard curve method.

### Mucin Histochemistry

Cultured airway cells were fixed with cold neutral buffered formalin for 60 min, then permeable supports stabilized in HistoGel (Richar-Allan Scientific, Kalamazoo, MI) before paraffin embedding. Thin sections from each filter were stained with H&E and PAS, and the number of PAS positive cells quantified by inspection by a single-blinded investigator. Fifty cells minimum were assessed per exposed filter. For MUC5ac antibody staining, slides were processed for antigen retrieval in pH 9.0 buffer (10 mM Tris, 1 mM EDTA), boiled for ten minutes at 15 PSI, then washed with 3% aqueous hydrogen peroxide for 5 min to quench endogenous peroxidase and afterwards rinsed with Tris buffer. Three percent goat serum was added for 20 min to block non-specific binding, then slides were incubated with monoclonal anti-MUC5ac antibody (1–13 M1,Neomarker, CA) diluted 1∶200 in PBE (1% BSA and 1 mM EDTA,in PBS at pH 7.6) buffer overnight in a humidity chamber at 4**°**C. Slides were then incubated at 25**°**C for 20 min with anti-mouse HRP secondary antibody (1∶200, Jackson Immuno Research) in PBE buffer. The slides were rinsed with Tris buffer and diaminobenzidine tetrachloride supersensitive substrate kit (Bio-Genex) was used to visualize the HRP reaction. Slides were counterstained with weak Mayer’s hematoxylin solution for 2 min. Negative control slides were prepared without addition of primary antibody.

### Cellular cAMP Measurements

Cellular cAMP was measured using an ELISA-based detection kit (Cayman Chemicals, Ann Arbor, MI) as previously described [22], [23]. Briefly, fully differentiated airway cells were stimulated with forskolin or vehicle control for 10 min, and cellular cAMP was extracted with ice-cold ethanol. The supernatants were vacuum dried, resuspended in phosphate buffer, and cAMP levels quantified per manufacturer’s directions.

### Mucus Transport Studies

HBE cells were washed with sterile PBS twice 1–2 days prior to addition of PEG beads. Fifty (50) µl of Diamine polyethylene glycol (PEG) coated fluorescent beads (1 µm, Molecular Probes, Eugene, OR, 1∶500 dilution in PBS) were added to the apical surface by using a microsprayer aerosolizer (Penn-Century IncModel IA-1B, Wyndmoor, PA). Following 24 h incubation, baseline images were obtained and then test compounds added to the basolateral compartment. Mucociliary transport (MCT) images were captured by time-lapse fluorescence imaging at four regions of interest per well located 1 mm from the periphery of the well and at each quadrant using an inverted epifluorescence microscope (Nikon Diaphot, Melvin, NY; 488 nm excitation/519 nm emission). Linear transport rates were computed using Metamorph 7.0 by analyzing 10–15 particles per region [24].

### Ciliary Beat Frequency (CBF) Analysis

HBE cells were allowed to equilibrate to room temperature for 15 min, then monitored by Hoffman contracts microscopy at 4–5 ROI for each well using high-speed digital video imaging at 100 frames/s, as previously described [25]. CBF was detected using Sisson-Avon Video Analysis (SAVA, Ammons Engineering, Mt. Morris, MI). Change in CBF was calculated as the change from baseline CBF detected in the same well 24 hours prior to addition of experimental conditions, normalized for the change in CBF observed with vehicle.

### Airway Surface Liquid (ASL) Depth Measurement by Confocal Microscopy

The apical surfaces of HBE cells were washed three times and then test compounds added to the basolateral compartment 24 h prior to labeling. Cells were stained with CMFDA (100 µM) in the cell culture medium for 1 hr. Texas red dye (25 µl at 10 mg/ml in Fc70) was added apically and cells allowed to equilibrate 2 h at 37°C. Transwell membranes were placed in sterile glass bottom dish coated with MEM, and imaged with a Carl Zeiss (Peabody, MA) confocal microscope using 20× (numerical aperture 0.88, working distance 0.55 mm) air objective lens. Cells were visualized with DIC optics to evaluate cell morphology before initiating fluorescence microscopy. Subsequently, Z-scan confocal fluorescent microscopy images were acquired from the top of the ASL through the top of the cell surface. XZ scans were analyzed using Zen2008 software at four ROI per well each located 1 mm from the filter periphery and at each quadrant; 5 estimates of ASL depth were taken equally dispersed across each ROI [24], [26]. Because baseline ASL depth varies among donors, each experiment was internally controlled using cells derived from a single donor.

**Figure 1:**
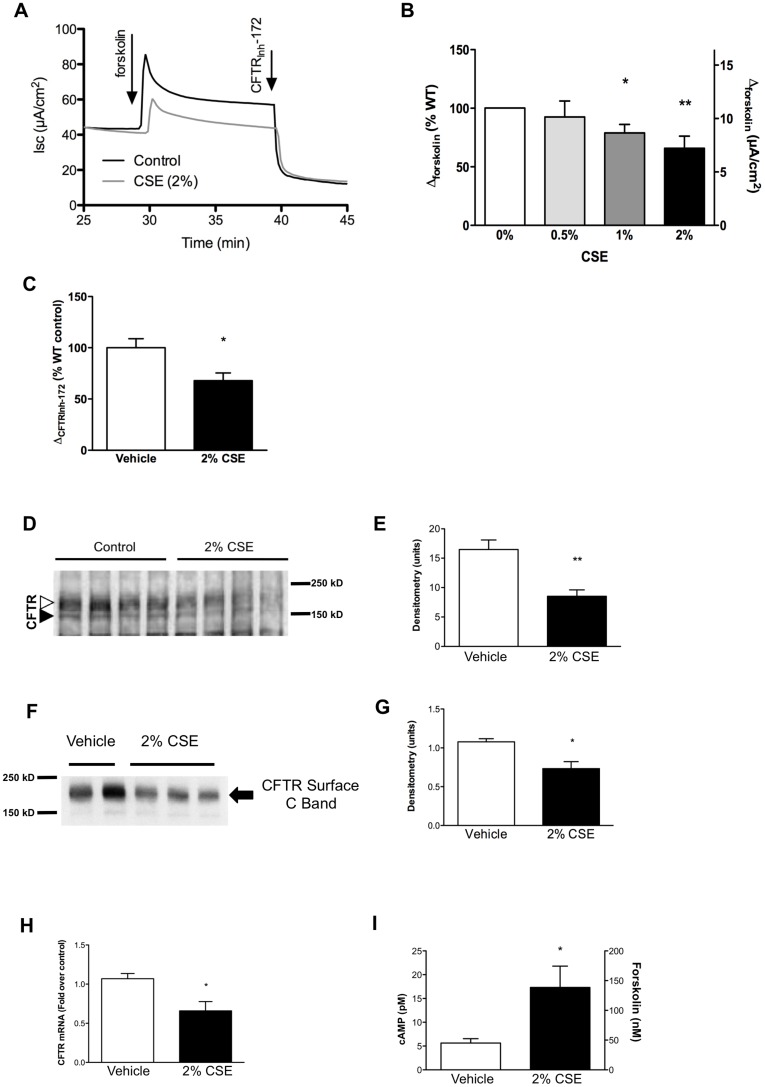
Cigarette smoke reduces CFTR-dependent activity and expression. (**A**) Representative tracings from primary human bronchial epithelial cells were grown at air-liquid interface until fully differentiated, followed by exposure to cigarette smoke extract (CSE 2%) on the apical surfaces for 24 h. Cells were then mounted in modified Ussing chambers and sequentially exposed to forskolin (20 µM) and CFTR_Inh_-172 (10 µM) in the setting of amiloride (100 µM) and Cl^–^free gluconate on the apical compartment. (**B–C**) Summary data of experiments shown outlined in (A). Change in short-circuit current (Isc) following addition of forskolin is shown as a percentage of wild-type non-CF current and absolute Isc (B). Change following 2% CSE is also shown for CFTR_Inh_-172 (10 µM, C). *P<0.05, **P<0.005, *n = *4 per concentration. (**D**) Western blot of cell lysates of primary HBE cells shown in (A) harvested immediately after treatment in the Ussing chamber. CFTR bands B and C are shown by the black and white arrows, respectively. All lanes were normalized for protein concentration and no difference was observed in protein levels at baseline: vehicle control treated wells (1.79±0.37 mg/ml) vs. CSE treated wells (1.92±0.40 mg/ml; P = 0.81); α-tubulin is also shown as a loading control. This blot is representative of 3 similar experiments. (**E**) Densitometry of CFTR band C shown in (D). **P<0.01. (**F**) Surface CFTR expression was quantified by a cell surface biotinylation assay following CSE (2%) or vehicle control treatment for 24 hrs. Blot is representative of 3 similar experiments. (**G**) Densitometry of surface CFTR band shown in (F). *P<0.05. (**H**) Primary HBE cells were exposed to CSE (2%) 24 h, then RNA isolated, and quantitative RT-PCR performed in comparison to 18S RNA. *P<0.05, *n = *9/condition. (**I**) cAMP concentration in HBE cells exposed to CSE (2%) or vehicle control for 24 h prior to assay. Equivalent forskolin concentration was calculated based on a standard curve in the same cells (See Fig. S2). *P<0.05.

### Nasal Potential Difference Measurements in Human Subjects

NPD protocols were approved by the University of Alabama at Birmingham’s IRB, and all subjects provided written informed consent. Inclusion criteria required age 40–80 and no respiratory illness in the last month that required antibiotics or steroids. A minimum of 10 pack-years tobacco use was required for all COPD patients, and current smokers were defined as smoking at least 10 cigarettes daily. Former smokers must have been abstinent for ≥1 year confirmed by measurement of urine cotinine. COPD patients must also have had a post-bronchodilator FEV_1_/FVC ratio <70% and an FEV_1_ between 40 and 70% predicted. Exclusion criteria include asthma or other lung disease or a change in medications one month prior to enrollment. The presence and severity of bronchitis was assessed by the Breathless Cough and Sputum Score (BCSS) as described by Leidy et al [27].

NPD studies were performed based on the description by Solomon et al.[28] and the Standard Operating Procedure of the CF-Therapeutics Development Network (Non-Perfusion method; SOP 528.00) using Electronic Data Capture (AD Instruments, Colorado Springs, CO), KCl calomel electrodes (Fischer Scientific, Pittsburgh, PA) and 3% agar nasal catheter and reference bridges.

**Figure 2:**
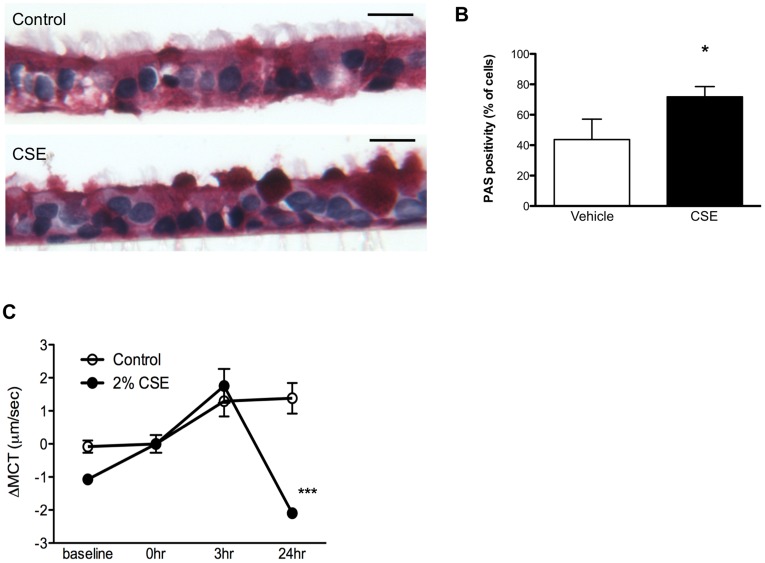
Mucociliary transport is inhibited by CSE. (**A**) Representative photomicrograph of fully differentiated HBE cells exposed to CSE (2%, apical) or vehicle control for 24 h then fixed, sectioned, and stained with PAS. PAS staining of intracellular and extracellular mucus shows as dark red. Scale bar depicts 25 µm. (**B**) Summary data of proportion of PAS positive cells from slides shown in (A). *P = 0.05, *n = *7–10/condition. (**C**) HBE cells were exposed to CSE (2%) or vehicle control, and transport rates of 1 µm fluorescent particles monitored. Baseline measures were made 24 h preceding time 0 h, followed by application of CSE or vehicle, then monitored at the times indicated. All results are normalized to transport rate at time 0 h for each individual well. *P<0.05, **P<0.001, *n = *10 particles/well.

### CFTR Genetics

CFTR genetic testing (50 mutation analysis) was performed on all airway tissue samples and NPD participants at a commercially accredited facility (Baylor Medical Genetics, Houston, TX), and included the 23 mutations recommended by the American College of Medical Genetics (ACMG) and reflex testing of the 5T allele upon detection of the R117H mutation.

### Reagents

Forskolin (Calbiochem; San Diego, CA), ATP (Sigma-Aldrich), vasoactive intestinal peptide (Sigma-Aldrich), CFTR_Inh_-172 (Calbiochem), and ivacaftor (Exclusive Chemistry, Obninsk, Russia) were used for experiments. Organically soluble cigarette smoke extract (CSE) was prepared by bubbling smoke from 1 2R4 research cig/ml DMSO (University of Kentucky, Lexington, KY) at 2 sec/10 ml puff/over 3 min to make 100% extract (OD_320_ = 0.2 at 100-fold dilution), then applied to cells diluted in media at the percentages shown, as previously described [29]. A volume of 25 µL CSE in media was applied to 0.33 cm^2^ transwell filters, and the exact same volume of media with DMSO vehicle was applied to controls. CSE at this concentration is thought to resemble exposures of cigarette smoke experienced in moderately heavy smokers [30], [31], [32], including the organically soluble form.

### Statistics

For Isc, cAMP measurements, Western blot densitometry, mucus transport rates, cilia beat frequency, and airway surface liquid depth, and potential difference measurements, descriptive statistics (mean, SD, and SEM) were compared using Student’s t-test or ANOVA, as appropriate. Post-hoc tests for multiple comparisons following ANOVA were calculated using Fisher’s least significant difference. All statistical tests were two-sided and were performed at a 5% significance level (i.e., á = 0.05) using GraphPad Prism (La Jolla, CA). Error bars designate SEM unless indicated otherwise. Correlation analysis was performed using SPSS (IBM, Armonk, NY).

## Results

To establish that exposure to cigarette smoke inhibits CFTR-dependent ion transport in our model, we exposed the apical surfaces of fully differentiated primary airway epithelial cells (HBE) derived from non-CF (wild-type) donors to increasing concentrations of organically soluble CSE, mimicking exposure levels in cigarette smokers [30], [31], [32]. As shown in Fig. 1A–C, CSE reduced the cAMP-dependent current in a dose-dependent fashion and at concentrations that did not alter transepithelial resistance (133.1±11.1 Ω⋅cm^2^ (control) vs. 168.7±15.6 (CSE 2%), P = 0.84). Changes in CFTR activity were accompanied by reduced delivery of CFTR to the cell surface, demonstrated by a reduction in the steady-state levels of fully-glycosylated CFTR (mature C band) compared to untreated controls (Fig. 1E,E) and cell-surface CFTR determined by a biotinylation assay (Fig. 1F,G). Similarly, CFTR mRNA transcript levels were reduced in CSE-treated monolayers compared to untreated controls assessed by real-time RT-PCR (Fig. 1H). The effects of CSE on ion transport were confirmed in Calu-3 cells, a model of airway serous glandular epithelium that exhibits high levels of endogenous wild-type (WT)-CFTR expression (Fig. S1A,B). Cyclic AMP levels were modestly elevated in HBE cells exposed to CSE for 24 h, establishing that reduced cAMP did not underlie the deleterious effects on CFTR activity. Cyclic AMP levels induced by CSE were equivalent to concentrations observed following addition of 130 nM forskolin based on a standard curve (Fig. 1I and Fig. S2). These results suggest that CSE reduced CFTR-dependent anion transport in part due to reduced delivery of CFTR to the cell surface resulting in deficient epithelial anion transport, thus extending prior reports with CSE [13], [14] and gas phase cigarette smoke [33].

**Table 1:** Demographics of Study Subjects.

	Non-smokers without COPD	Non-smokers with COPD	Smokers with COPD	Smokers without COPD
	(*n = *8)	(*n = *12)	(*n = *12)	(*n = *12)
Age				
Mean	57	66.7	55.6	51
Range	45–74	66–77	49–66	47–57
Gender, Female				
* n* (%)	5 (62.5)	6 (50)	5 (42)	5 (42)
Smoking history – pack years				
Mean	0	48.2	55.1	33.2
Range	0	35–80	35–78	10–78
FEV_1_/FVC, mean	0.80	0.50	0.53	0.79
FEV_1_% predicted				
Mean	99	56.9	54.1	92.2
Range	74–123	43–73	32–82	76–114

**Figure 3:**
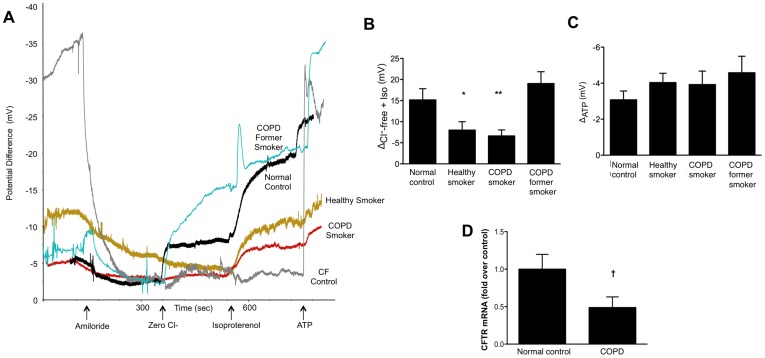
Reduced CFTR activity in smokers with COPD measured by potential difference. (**A**) Representative nasal potential difference (NPD) tracings from a normal subject, a smoker with COPD, a COPD subject who no longer smokes and a healthy smoker are shown. Nasal membrane was sequentially perfused with Ringer’s solution, Ringer’s plus amiloride (100 µM), Cl^–^free gluconate with amiloride, Cl^–^free gluconate with amiloride plus isoproterenol (100 µM), and Cl^–^free gluconate with amiloride plus isoproterenol and ATP (10 µM). A CF subject is shown for comparison. (**B–C**) Summary data derived from subjects shown in (A) demonstrating CFTR-dependent Cl^−^ conductance (Δ_Cl_^–^_free_ _+ Iso_, B) and ΔPD following ATP perfusion (C). *P<0.05, **P<0.005. (**D**) CFTR mRNA expression determined by real-time RT-PCR and normalized to 18S endogenous control. Levels are expressed relative to mean mRNA level in normal subjects. **^τ^**P = 0.07, *n = *16, 13.

Given the deleterious effects of CSE on CFTR-dependent anion conductance, we next examined the impact of CSE exposure on mucus expression and mucociliary transport. CSE exposure generated a pronounced increase in mucus expression measured by histologic staining of PAS positive material (Fig. 2A,B). MUC5ac was highly expressed in CSE-exposed HBE cells, similar to the phenotypic expression in the bronchi of human smokers (Fig. S3) [34]. The changes in CFTR activity and mucus expression induced by CSE were associated with prominent reductions in mucociliary transport rates measured by fluorescence microscopy (Fig. 2C).

Given the effects of CSE on CFTR-dependent ion transport and mucus transport in respiratory epithelial cells, we next assessed whether this defect is also detectable *in vivo* among individuals with smoking related COPD. While reduced CFTR activity has been previously reported in healthy smokers without CFTR mutations, individuals with COPD have not been studied [13]. Because ion transport defects in primary respiratory epithelial cells are highly predictive of ion transport *in vivo* [16], [17], [35], we hypothesized that individuals with smoking-related COPD would be characterized by reduced CFTR expression and activity measured by nasal potential difference (NPD). We enrolled individuals with COPD who currently smoke and compared CFTR-dependent chloride transport to results observed in age-matched healthy controls, smokers without airflow obstruction, and COPD subjects who had quit smoking for at least 1 year (Table 1). One healthy non-smoker control was identified as a CF carrier, and was excluded from the analysis. Smokers with COPD exhibited a severe reduction in CFTR-dependent ion transport (Fig. 3A,B) that was slightly lower than healthy smokers; the chloride conductance of former smokers with COPD was not affected (Fig. 3A,B), indicating that CFTR function can recover following prolonged smoking cessation. Baseline voltage was 7.7±0.8 in COPD smokers compared to 13.6±2.4 in healthy controls (P<0.05) and amiloride sensitive PD was also reduced in COPD smokers, suggesting that unlike CF, smoking is not associated with baseline hyperpolarization of the nasal membrane. The defect in chloride conductance from smoking was not attributable to changes in mucosal integrity and appeared specific to CFTR, as no significant difference in PD was observed following adenosine triphosphate (ATP) perfusion, which stimulates activity of Cl^−^ channels other than CFTR (Fig. 3C). We also observed a specific reduction in CFTR mRNA expression in nasal curettage samples obtained from individuals with COPD (Fig. 3D). Reduced CFTR activity measured by NPD was also associated with the severity of bronchitic symptoms (r = 0.30, P<0.05), as assessed by BCSS [27], even when controlled for cigarette smoking (r = 0.31, P<0.05), indicating an association with a phenotype reminiscent of CF (i.e. chronic bronchitis).

**Figure 4:**
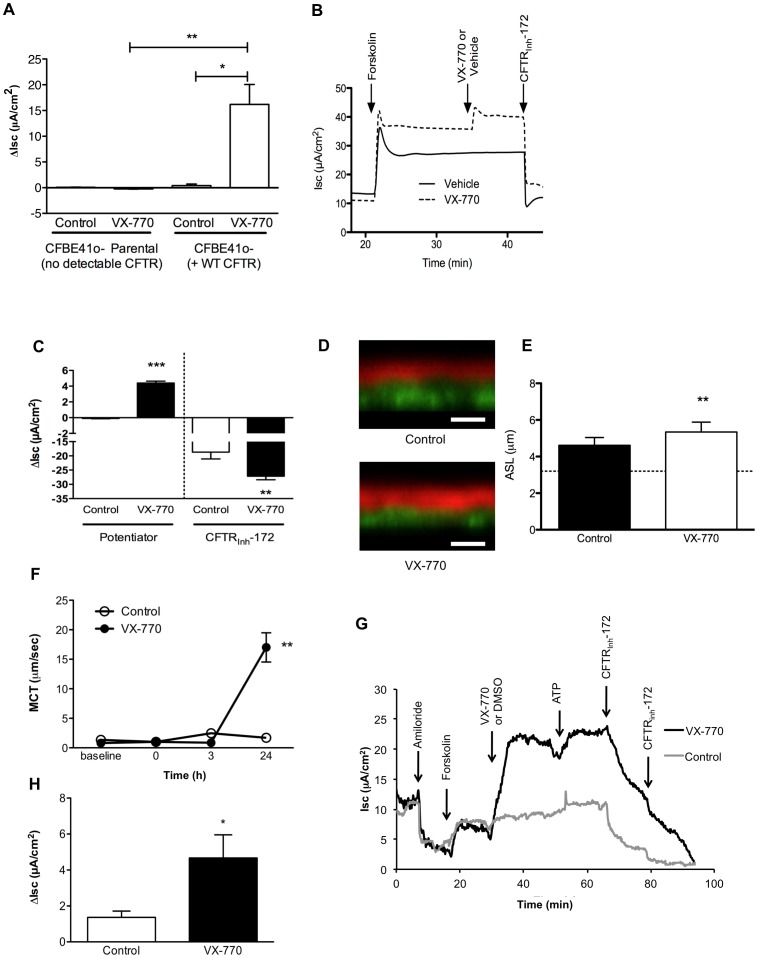
The CFTR potentiator ivacaftor augments wild-type CFTR anion transport resulting in enhanced mucus transport. (**A**) CFBE41o- cells were grown at air-liquid interface, then mounted in Ussing chambers and stimulated with ivacaftor (10 µM) or vehicle control following amiloride (100 µM) and forskolin (100 nM) in the setting of a Cl^−^ secretory gradient. CFBE41o- cells complemented by stable expression of WT-CFTR (by a lentivirus promoter) are shown in comparison to CFBE41o- cells without WT CFTR complementation (parental cells). *P<0.05, **P<0.005, n = 5/condition. (**B**) Representative Isc tracings of primary HBE cells sequentially exposed to forskolin (100 nM), ivacaftor (10 µM) or vehicle control, followed by CFTR_Inh_-172 (10 µM) in the setting of amiloride (100 µM) and a chloride secretory gradient. (**C**) Summary data of tracings shown in (B) indicating stimulated Isc following acute addition of ivacaftor. ** P<0.005, ***P<0.0005, *n* = 5–6/condition. (**D**) Representative Z-scan confocal images derived from HBE cells following exposure to vehicle control or ivacaftor (10 µM) to the basolateral compartment for 24 h prior to assay. White scale bars designate 10 µm. (**E**) Summary data from experiments shown in (D). **P<0.005, *n = *10/condition. (**F**) Mucociliary transport rates derived from HBE cells. ivacaftor (10 µM) or vehicle control was added to basolateral compartment of monolayers co-stimulated with the cAMP agonist VIP (30 nM) immediately after the time  = 0 h measurement. **P<0.005 vs. vehicle control, *n* = 10 particles/condition. (**G**) Representative Isc tracing of bronchus derived from a normal human subject following dissection of the mucosal layer that was then mounted in an Ussing chamber under voltage clamp conditions and bathed in symmetric Ringer’s solution. Serial addition of amiloride (100 µM), forskolin (100 nM), and ivacaftor (10 µM) or vehicle control is shown, followed by addition of ATP (10 µM), and CFTR_Inh_-172 (10 µM×2) as control additions. (**H**) Summary data derived from experiments shown in (G). The change in Isc following addition of ivacaftor or vehicle control is shown. *P<0.05, *n* = 19, 24 samples/condition.

Recently the potentiator ivacaftor was reported to significantly augment cAMP mediated ion transport activity of CFTR encoding the gating mutation, G551D-CFTR, *in vitro* [17] and in CF subjects harboring the G551D CFTR defect [16]. Ivacaftor improved measures of CFTR activity (including both chloride conductance monitored by NPD and sweat chloride [16]) and also produced a marked and sustained improvement in pulmonary function in CF patients with G551D-CFTR [15], [16], establishing that mutant CFTR is a valid therapeutic target in CF. To assess whether ivacaftor also promotes the anion channel activity of WT-CFTR, which could thereby serve as a therapeutic target in acquired disorders of CFTR function, we examined the activity of ivacaftor in WT-CFTR expressing cells. Ivacaftor induced robust increases in anion transport in CFBE41o- cells complemented with stable WT-CFTR expression, whereas no activity was observed in parental cells without detectable CFTR expression, establishing specificity for CFTR (Fig. 4A). The increase in anion transport by ivacaftor was related to a pronounced increase in the open probability of WT-CFTR expressed in NIH 3T3 cells (Fig. S4) as measured by single channel studies using an inside-out membrane patch configuration. In primary non-CF HBE cells, ivacaftor also augmented CFTR-dependent anion transport activity following pre-stimulation with 100 nM forskolin, a dose chosen to induce cAMP levels matching CSE-exposed cells, increasing CFTR-dependent short-circuit current (Isc) stimulated by forskolin alone (Fig. 4B,C). The activity of ivacaftor in WT-CFTR expressing cells varied according to the degree of forskolin pre-stimulation in HBE cells (Fig. S5), and was maximal at concentrations approximating 10–100 nM forskolin. The increase in CFTR function by ivacaftor was associated with increased ASL depth of non-CF HBE monolayers following 24 h treatment to the basolateral compartment, reflecting augmented CFTR-dependent fluid transport (Fig. 4D,E). While ivacaftor induced a modest but significant increase in ASL depth, the effects on MCT were dramatic at 24 h (Fig. 4F). Ivacaftor also potentiated CFTR-dependent current in normal explanted human trachea examined under voltage clamp conditions (Fig. 4G,H); the effects of ivacaftor were similar (P = NS) whether the individual was an active smoker (6.8±5.6 µA/cm^2^) or non-smoker (9.9±3.4 µA/cm^2^), suggesting the therapeutic potential of ivacaftor in individuals with a history of smoking.

**Figure 5:**
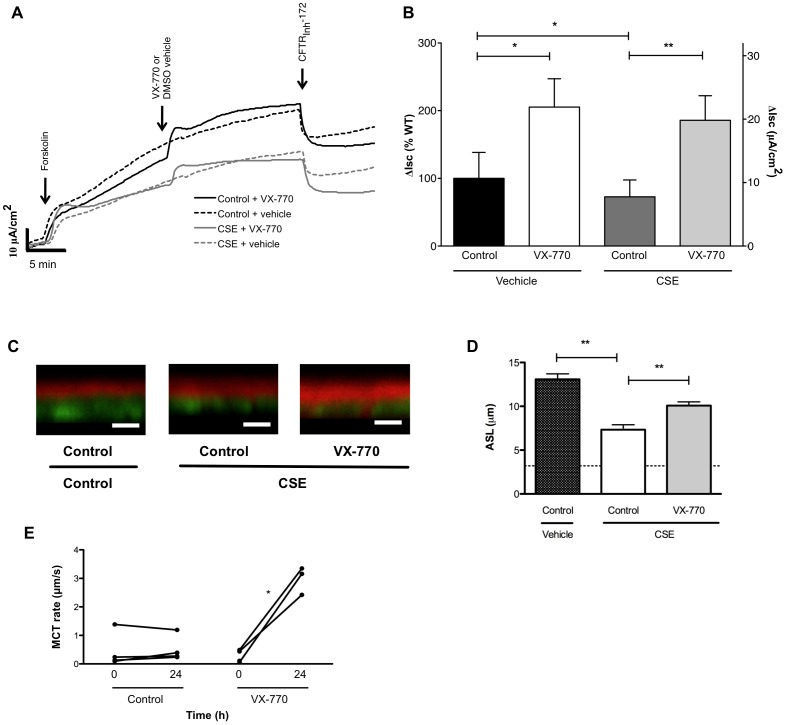
The CFTR Potentiator ivacaftor rescues block of CSE-induced CFTR-dependent epithelial function. (**A**) Primary HBE cells were treated with 2% CSE or vehicle control for 24 h, then studied under voltage clamp conditions. Cells were stimulated with forskolin (100 nM), ivacaftor (10 µM) or vehicle control, and then CFTR_Inh_-172 (10 µM) to block CFTR. (**B**) Summary data of experiments shown in (A). Total stimulated Isc (forskolin + ivacaftor or vehicle control) is shown as the percentage of wild-type Isc with forskolin (100 nM) alone, *n* = 6 per condition. (**C**) Representative Z-scan confocal images derived from HBE cells following exposure to CSE (2%, apical), CSE + ivacaftor (10 µM basolateral), or vehicle control for 24 h prior to assay. White scale bars designate 10 µm. (**D**) Summary data from experiments shown in (C). **P<0.005, *n* = 15/condition. Dotted line indicates ASL depth of a panel of CF HBE cells using the exact same method. (**E**) Mucociilary transport studies were performed in HBE cells using movement of fluorescent beads 1 mm from the filter periphery. Beads were added 24 h prior to time 0 h measurements, then CSE (2%) was added to the apical compartment in addition to ivacaftor (10 µM) or vehicle control to the basolateral compartment. *P<0.05.

Given the robust effects of ivacaftor in non-CF epithelia, including potentiation of CFTR-mediated ion transport, ASL depth, and MCT, we next examined whether CSE mediated CFTR inhibition could be overcome by application of ivacaftor, and the resultant effects on ASL and MCT. Ivacaftor potentiated CFTR-dependent Isc, regardless of prior administration of CSE (Fig. 5A,B). Ivacaftor partially restored depletion of ASL depth in CSE treated monolayers (Fig. 5C,D) and also caused a significant increase in MCT rate (Fig. 5E), though ciliary beating was only minimally affected (Fig. S6).

**Figure 6:**
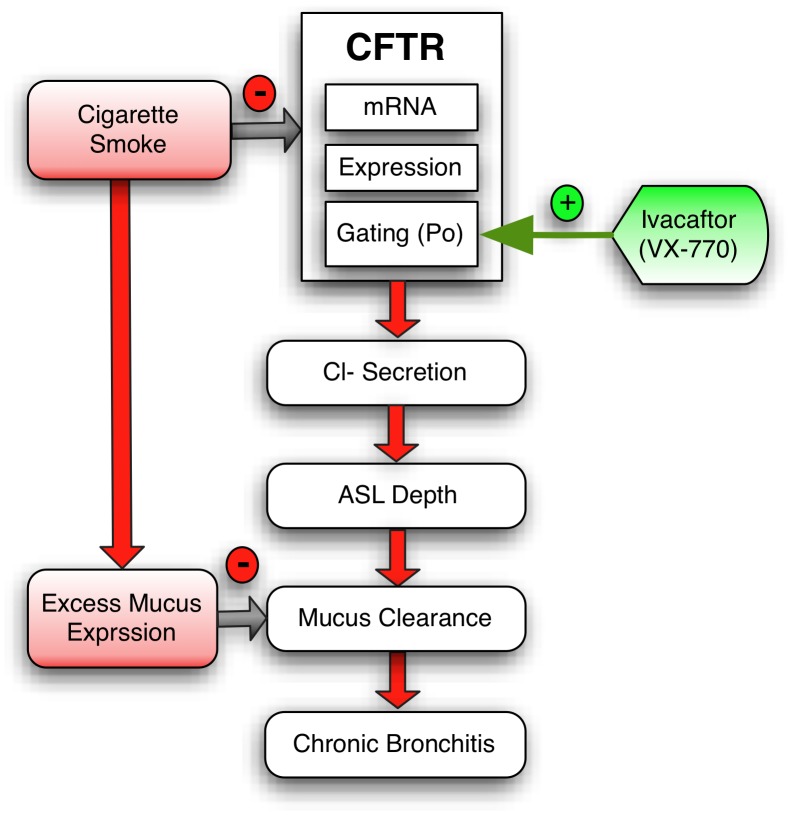
Schematic demonstrating the role of cigarette smoke and CFTR potentiator therapy in chronic bronchitis.

## Discussion

We demonstrate that acquired CFTR dysfunction induced by cigarette smoke contributes to mucus stasis, an important predictor of morbidity and mortality in COPD [10], [12], and that this abnormality is markedly improved by the CFTR potentiator ivacaftor *in vitro*. Moreover, the results provide a strong basis to consider CFTR potentiator treatment as a pharmacologic strategy to directly address acquired CFTR dysfunction in COPD using a treatment already available for CF.

The deleterious effects of cigarette smoke exposure on anion transport in respiratory epithelia were first observed in dog trachea by Welsh prior to CFTR gene discovery [36]. Kreindler et al. implicated CFTR when demonstrating that CSE altered chloride and potassium conductance in primary non-CF HBE cell cultures [14], findings later confirmed with gas phase cigarette smoke by Savitski et al. [33]. These data also support Cantin et al. who demonstrated that CFTR mRNA, protein expression and ion transport were reduced in Calu-3 cells following CSE exposure *in vitro* and that chloride transport was impaired in NPD testing of healthy smokers without CFTR mutations [13]. Our observations regarding the effects of cigarette smoke on CFTR-mediated ion transport now include smokers with COPD. The downstream effects of cigarette exposure on mucus expression and transport shown here are natural consequences of an altered ion transport milieu and form the basis for evaluating compounds that target CFTR activation. Although former smokers with COPD largely recover CFTR activity as assessed by NPD, we do not yet know whether CFTR function normalizes in the lung, where additional insults may also contribute to CFTR deficiency. Regardless, our results indicate that individuals with sustained CFTR dysfunction are more predisposed to symptoms attributable to delayed mucociliary transport, including the severity of daily chronic cough independent of current cigarette smoking.

Our findings provide a clear indication that cigarette smoke exposure and the associated decrease in CFTR function is causally related to decreased mucociliary transport observed in smokers with COPD. When the effect of cigarette smoke on CFTR-dependent fluid secretion is accompanied by augmented mucus expression, MCT is markedly decreased (Fig. 6). Our findings demonstrate the importance of the fluid secretion/mucus expression balance required to regulate mucus clearance, a hypothesis previously postulated by Kreindler [14]. Measurements of transepithelial resistance, direct visualization following fixation, immunohistochemistry, CFTR-dependent ion transport stimulation, ASL depth measurements and fluorescent particle mobility confirm the assertion that the effects of CSE are mediated through CFTR-dependent fluid secretion, and not simply due to disruption of the integrity of cell monolayers.

An improved understanding of the role of CFTR in the maintenance of normal epithelial function has revealed that mild/variable CFTR mutations also play a causative role in a number of diseases not classically associated with CF. For example, mild/variable CFTR mutations are present in ∼30% of individuals with recurrent idiopathic pancreatitis [37], [38], and similar associations have been established in congenital bilateral absence of the vas deferens [39], allergic bronchopulmonary aspergillosis [40], [41], chronic sinusitis [42], and idiopathic bronchiectasis [43], [44]. The basis of these diseases illustrate that mild CFTR dysfunction can contribute to substantial pathology, even among individuals not previously recognized to have dysfunctional ion transport [45]. Although we observed only a partial inhibition of CFTR activity following cigarette smoke exposure *in vivo* or *in vitro*, the functional decrement is equivalent to that observed in individuals with mild/variable CFTR mutations associated with clinical CF and significantly greater than asymptomatic heterozygotes [46]. Combined with increased mucus expression expected following cigarette smoking (and observed in our studies), we posit that these abnormalities are sufficient to induce a severe abnormality in MCT in the lung and contribute to mucus stasis in the small airways, resulting in symptoms of chronic bronchitis. Delayed MCT through this pathway may also be responsible for reduced MCC detected in individuals with chronic bronchitis in comparison to COPD patients without chronic mucus expectoration [47]. The robust response of MCT to the application of the CFTR potentiator ivacaftor, an agent with a high degree of selectivity for CFTR [17], further strengthens the argument that deficient CFTR activity has an important role governing delayed MCC in smoking-related lung diseases (Fig. 6).

Because animal models of smoking induced bronchitis that also exhibit lung disease attributable to CFTR mutations are not available (e.g., mice do not exhibit CF lung disease nor bronchitis), the present findings were largely focused on the use of primary bronchial epithelial cell cultures, complemented by evaluation of intact tissues and *in vivo* measures in human subjects. An important strength of the HBE model is that it is highly representative of the ion transport environment in the human airway [24] and predictive of pharmacologic benefit with CFTR modulators in CF [17]. This relationship is borne out by our studies, which also demonstrate a close resemblance between *in vitro* and *in vivo* measurements of ion transport. Though the HBE model faithfully replicates in vivo findings in CF, we do not yet have evidence that ivacaftor augments CFTR activity *in vivo* in individuals without CF. Moreover, our experimental model relies on acute exposure to cigarette smoke, and cannot account for the myriad of pathophysiologic pathways and compensatory mechanisms that are induced with chronic smoking and may impact MCT, including the effects of squamous metaplasia, glandular hypertrophy, or subepithelial inflammation. Of note, the effect of ivacaftor on MCT rates was disproportionately large compared to its effect on ASL depth or ciliary beating, suggesting that the MCT apparatus is highly sensitive to fluid secretion. We do not yet know whether the improvement in MCT may be in part due to changes in mucus viscosity, adherence, or relative sensitivity of mucosal transport to fluid secretion. It is also possible that ivacaftor may lead to reduced mucin expression which could contribute to these robust effects, though this was not reported in preclinical testing of the compound.

Our studies show that reduced CFTR function caused by smoking was not associated with increased sodium transport, as commonly observed in CF. Rather, reduced CFTR mediated anion secretion appears to be the major cause of reduced ASL depth induced by smoking, though other ion transport pathways not measured by our studies could also contribute. Notably, a trend towards reduced sodium conductance was also reported by Cantin and Kreindler (12, 13) supporting our findings and highlighting that the changes in epithelial function caused by smoking are more complex than those observed when CFTR is genetically absent. Regardless of the relative contributions of other ion transport pathways, the robust effects of ivacaftor on ASL depth and MCT rates strongly indicate that increased chloride transport is sufficient to augment epithelial function and provide a compelling rationale to consider testing CFTR potentiators in individuals with smoking-related COPD, regardless of the status of sodium transport.

Other noxious stimuli particularly relevant to the COPD lower airway have recently been postulated to reduce CFTR activity, and may contribute to the *in vivo* deficit. Heavy metals, such as cadmium, have been proposed to be responsible for deleterious effects of cigarette smoke on CFTR expression [48]. Guimbellot et al. showed that hypoxia in the airways led to reduced CFTR mRNA expression and activity *in vitro*, and reduced CFTR mRNA expression was also detected in mice exposed to hypoxic conditions [49]. Rab et al. demonstrated that pro-inflammatory environments, such as those expected in the COPD lung, induce the unfolded protein response, and thus reduce CFTR function [50]. Accumulation of ceramide due to the UPR was also observed in the lungs of COPD patients and was correlated with reduced CFTR expression in those tissues [51], [52]. Further studies are needed to establish the relative contribution of these molecular mechanisms to acquired CFTR dysfunction in the COPD lung and to determine if they might contribute to persistent decrements in CFTR activity following smoking cessation.

Though these findings point to a novel therapeutic strategy for COPD, additional investigations are needed prior to implementing such a treatment paradigm. While our studies convincingly demonstrate that a CFTR decrement is present in the upper airway of smokers with COPD, CFTR function has not yet been measured in the lower airway of these patients. In addition, the relationship between lower airway CFTR activity and small airway mucus clearance is unknown as is the effect of CFTR dysfunction on clinical phenotype (e.g. exacerbation frequency, lung function, and symptoms of bronchitis and dyspnea). Establishing the phenotypic groups in which CFTR dysfunction leads to COPD manifestations is critical, since application of CFTR potentiator therapy may be prohibitively expensive for broad non-targeted use.

CFTR potentiators such as ivacaftor represent an attractive therapeutic approach for individuals with smoking related lung disease and may be particularly well suited to individuals with chronic mucus hypersecretion. As opposed to currently available mucolytic agents that have failed to demonstrate convincing evidence of clinical benefit [53], [54], CFTR potentiators may overcome the limitations posed by the poor bioavailability of pharmaceuticals administered by inhalation and the limited activity of oral agents none of which directly augment MCC. Because mucus stasis has been associated with excess mortality and a more rapid decline in pulmonary function [10], this remains an area of high therapeutic priority in COPD [55]. Further studies to establish the phenotype associated with CFTR dysfunction among individuals with COPD are indicated, and together with the present findings, could form the basis for clinical intervention using CFTR potentiators in COPD.

## Supporting Information

Figure S1 **(A)** Representative Ussing chamber tracings from Calu-3 cells grown at air-liquid interface, then exposed to CSE (2%) for 24 h and studied under voltage clamp conditions as shown in Fig. 1A. **(B)** Summary data of experiments shown in (A). ΔIsc is shown following stimulation with forskolin (20 µM) *P<0.05, *n* = 8 **P<0.01, *n* = 8.
(TIFF)

Figure S2 Cellular cAMP levels determined by colorimetric assay in non-CF HBE monolayers stimulated with forskolin (0, 100, 300 and 1000 nM) for 10 min prior to lysis, *n* = 3/condition.
(TIFF)

Figure S3 **MUC5ac expression is altered by CSE exposure.** MUC5ac staining is shown from representative sections of HBE cells exposed to CSE (2%) or vehicle control for 24 h. Control slides were stained in the absence of primary anti-MUC5ac antibody.
(TIFF)

Figure S4 **Effect of ivacaftor on open channel probability.** Inside-out membrane patches were obtained from NIH3T3 cells transduced with WT CFTR and single channel conductance tracings recorded with control (**i**, P_o = _0.4±0.04); 0.3 µM ivacaftor (ivacaftor; **ii**, P_o = _0.8±0.04); and 0.3 µM ivacaftor +20 µM CFTR_Inh_-172 (**iii**). All recordings performed in the presence of 75 nM PKA +1 mM ATP. Dotted line represents closed channel configuration. Summary data of these experiments was shown previously by Van Goor et al. [17].
(TIFF)

Figure S5 **Effect of forskolin prestimulation on ivacaftor induced Isc.** Prior to ivacaftor addition, varying concentrations of forskolin were administered as shown to non-CF HBE cells, then Isc plotted following sequential addition of increasing concentrations of ivacaftor (ivacaftor), *n = *3/condition.
(TIFF)

Figure S6 **Effect of CSE exposure on ciliary beating.** CSE (2%, apical), ivacaftor (ivacaftor; 10 µM, basolateral), both agents, or vehicle control was applied to primary human bronchial epithelial monolayers for 24 hrs, and then the change in ciliary beat frequency (CBF) from pretreatment baseline was assessed by Hoffman contrast microscopy at 4–5 ROI for each well. N = 3/condition.
(TIFF)

## References

[1] Rowe SM, Miller S, Sorscher EJ (2005). Cystic fibrosis.. N Engl J Med.

[2] Amaral MD, Kunzelmann K (2007). Molecular targeting of CFTR as a therapeutic approach to cystic fibrosis.. Trends Pharmacol Sci.

[3] Varga K, Goldstein RF, Jurkuvenaite A, Chen L, Matalon S (2008). Enhanced cell-surface stability of rescued DeltaF508 cystic fibrosis transmembrane conductance regulator (CFTR) by pharmacological chaperones.. Biochem J.

[4] Houghton AM, Quintero PA, Perkins DL, Kobayashi DK, Kelley DG (2006). Elastin fragments drive disease progression in a murine model of emphysema.. J Clin Invest.

[5] Kellermayer R, Szigeti R, Keeling KM, Bedekovics T, Bedwell DM (2006). Aminoglycosides as potential pharmacogenetic agents in the treatment of Hailey-Hailey disease.. J Invest Dermatol.

[6] Barnes PJ (2004). Small airways in COPD.. N Engl J Med.

[7] Martinez-Garcia MA, Soler-Cataluna JJ, Donat Sanz Y, Catalan Serra P, Agramunt Lerma M (2011). Factors associated with bronchiectasis in patients with COPD.. Chest.

[8] Sethi S, Maloney J, Grove L, Wrona C, Berenson CS (2006). Airway inflammation and bronchial bacterial colonization in chronic obstructive pulmonary disease.. Am J Respir Crit Care Med.

[9] Soler N, Ewig S, Torres A, Filella X, Gonzalez J (1999). Airway inflammation and bronchial microbial patterns in patients with stable chronic obstructive pulmonary disease.. Eur Respir J.

[10] Hogg JC, Chu F, Utokaparch S, Woods R, Elliott WM (2004). The nature of small-airway obstruction in chronic obstructive pulmonary disease.. N Engl J Med.

[11] Vestbo J (2002). Epidemiological studies in mucus hypersecretion.. Novartis Found Symp 248: 3–12; discussion 12–19, 277–282.

[12] Hogg JC, Chu FS, Tan WC, Sin DD, Patel SA (2007). Survival after lung volume reduction in chronic obstructive pulmonary disease: insights from small airway pathology.. Am J Respir Crit Care Med.

[13] Cantin AM, Hanrahan JW, Bilodeau G, Ellis L, Dupuis A (2006). Cystic fibrosis transmembrane conductance regulator function is suppressed in cigarette smokers.. Am J Respir Crit Care Med.

[14] Kreindler JL, Jackson AD, Kemp PA, Bridges RJ, Danahay H (2005). Inhibition of chloride secretion in human bronchial epithelial cells by cigarette smoke extract.. Am J Physiol Lung Cell Mol Physiol.

[15] Ramsey BW, Davies J, McElvaney NG, Tullis E, Bell SC (2011). A CFTR potentiator in patients with cystic fibrosis and the G551D mutation.. The New England Journal of Medicine.

[16] Accurso FJ, Rowe SM, Clancy JP, Boyle MP, Dunitz JM (2010). Effect of VX-770 in persons with cystic fibrosis and the G551D-CFTR mutation.. N Engl J Med.

[17] Van Goor F, Hadida S, Grootenhuis PD, Burton B, Cao D (2009). Rescue of CF airway epithelial cell function in vitro by a CFTR potentiator, VX-770.. Proc Natl Acad Sci U S A.

[18] Rowe SM, Dransfield MT (2010). Lower airway potential difference measurements in COPD subjects: Emerging evidence of acquired CFTR dysfunction.. Ped Pulmonol.

[19] Rowe SM, Pyle LC, Jurkevante A, Varga K, Collawn J (2010). DeltaF508 CFTR processing correction and activity in polarized airway and non-airway cell monolayers.. Pulm Pharmacol Ther.

[20] Jurkuvenaite A, Varga K, Nowotarski K, Kirk KL, Sorscher EJ (2006). Mutations in the amino terminus of the cystic fibrosis transmembrane conductance regulator enhance endocytosis.. J Biol Chem.

[21] Rowe SM, Varga K, Rab A, Bebok Z, Byram K (2007). Restoration of W1282X CFTR Activity by Enhanced Expression.. Am J Respir Cell Mol Biol.

[22] Bebok Z, Collawn JF, Wakefield J, Parker W, Li Y (2005). Failure of cAMP agonists to activate rescued deltaF508 CFTR in CFBE41o- airway epithelial monolayers.. J Physiol.

[23] Cobb BR, Fan L, Kovacs TE, Sorscher EJ, Clancy JP (2003). Adenosine receptors and phosphodiesterase inhibitors stimulate Cl^−^ secretion in Calu-3 cells.. Am J Respir Cell Mol Biol.

[24] Matsui H, Randell SH, Peretti SW, Davis CW, Boucher RC (1998). Coordinated clearance of periciliary liquid and mucus from airway surfaces.. Journal of Clinical Investigation.

[25] Azbell C, Zhang S, Skinner D, Fortenberry J, Sorscher EJ (2010). Hesperidin stimulates cystic fibrosis transmembrane conductance regulator-mediated chloride secretion and ciliary beat frequency in sinonasal epithelium.. Otolaryngology–head and neck surgery : official journal of American Academy of Otolaryngology-Head and Neck Surgery.

[26] Rollins BM, Burn M, Coakley RD, Chambers LA, Hirsh AJ (2008). A2B adenosine receptors regulate the mucus clearance component of the lung’s innate defense system.. Am J Respir Cell Mol Biol.

[27] Leidy NK, Rennard SI, Schmier J, Jones MK, Goldman M (2003). The breathlessness, cough, and sputum scale: the development of empirically based guidelines for interpretation.. Chest.

[28] Solomon GM, Konstan MW, Wilschanski M, Billings J, Sermet-Gaudelus I (2010). An international randomized multicenter comparison of nasal potential difference techniques.. Chest.

[29] van der Toorn M, Rezayat D, Kauffman HF, Bakker SJ, Gans RO (2009). Lipid-soluble components in cigarette smoke induce mitochondrial production of reactive oxygen species in lung epithelial cells.. Am J Physiol Lung Cell Mol Physiol.

[30] Sarir H, Mortaz E, Karimi K, Kraneveld AD, Rahman I (2009). Cigarette smoke regulates the expression of TLR4 and IL-8 production by human macrophages.. J Inflamm (Lond).

[31] Mortaz E, Kraneveld AD, Smit JJ, Kool M, Lambrecht BN (2009). Effect of cigarette smoke extract on dendritic cells and their impact on T-cell proliferation.. PLoS One.

[32] Kasai A, Hiramatsu N, Hayakawa K, Yao J, Maeda S (2006). High levels of dioxin-like potential in cigarette smoke evidenced by in vitro and in vivo biosensing.. Cancer Res.

[33] Savitski AN, Mesaros C, Blair IA, Cohen NA, Kreindler JL (2009). Secondhand smoke inhibits both Cl^−^ and K^+^ conductances in normal human bronchial epithelial cells.. Respir Res.

[34] Innes AL, Woodruff PG, Ferrando RE, Donnelly S, Dolganov GM (2006). Epithelial mucin stores are increased in the large airways of smokers with airflow obstruction.. Chest.

[35] Rowe SM, Clancy JP, Boyle M, Van Goor F, Ordonez C (2010). Parallel effects of VX-770 on transepithelial potential difference in vitro and in vivo.. Journal of Cystic Fibrosis 9 (Supplement).

[36] Welsh MJ (1983). Cigarette smoke inhibition of ion transport in canine tracheal epithelium.. J Clin Invest.

[37] Cohn JA, Friedman KJ, Noone PG, Knowles MR, Silverman LM (1998). Relation between mutations of the cystic fibrosis gene and idiopathic pancreatitis.. N Engl J Med.

[38] Sharer N, Schwarz M, Malone G, Howarth A, Painter J (1998). Mutations of the cystic fibrosis gene in patients with chronic pancreatitis.. N Engl J Med.

[39] Kerem E, Hirawat S, Armoni S, Yaakov Y, Shoseyov D (2008). Effectiveness of PTC124 treatment of cystic fibrosis caused by nonsense mutations: a prospective phase II trial.. Lancet.

[40] Marchand E, Verellen-Dumoulin C, Mairesse M, Delaunois L, Brancaleone P (2001). Frequency of cystic fibrosis transmembrane conductance regulator gene mutations and 5T allele in patients with allergic bronchopulmonary aspergillosis.. Chest.

[41] Miller PW, Hamosh A, Macek M, Greenberger PA, MacLean J (1996). Cystic fibrosis transmembrane conductance regulator (CFTR) gene mutations in allergic bronchopulmonary aspergillosis.. American journal of human genetics.

[42] Howard MT, Shirts BH, Petros LM, Flanigan KM, Gesteland RF (2000). Sequence specificity of aminoglycoside-induced stop condon readthrough: potential implications for treatment of Duchenne muscular dystrophy.. Ann Neurol.

[43] Pignatti PF, Bombieri C, Benetazzo M, Casartelli A, Trabetti E (1996). CFTR gene variant IVS8–5T in disseminated bronchiectasis.. Am J Hum Genet.

[44] Girodon E, Cazeneuve C, Lebargy F, Chinet T, Costes B (1997). CFTR gene mutations in adults with disseminated bronchiectasis.. Eur J Hum Genet.

[45] Knowles MR, Durie PR (2002). What is cystic fibrosis?. N Engl J Med.

[46] Wilschanski M, Dupuis A, Ellis L, Jarvi K, Zielenski J (2006). Mutations in the cystic fibrosis transmembrane regulator gene and in vivo transepithelial potentials.. Am J Respir Crit Care Med.

[47] Koblizek V, Tomsova M, Cermakova E, Papousek P, Pracharova S (2011). Impairment of nasal mucociliary clearance in former smokers with stable chronic obstructive pulmonary disease relates to the presence of a chronic bronchitis phenotype.. Rhinology.

[48] Rennolds J, Butler S, Maloney K, Boyaka PN, Davis IC (2010). Cadmium regulates the expression of the CFTR chloride channel in human airway epithelial cells.. Toxicological sciences : an official journal of the Society of Toxicology.

[49] Guimbellot JS, Fortenberry JA, Siegal GP, Moore B, Wen H (2008). Role of oxygen availability in CFTR expression and function.. Am J Respir Cell Mol Biol.

[50] Rab A, Bartoszewski R, Jurkuvenaite A, Wakefield J, Collawn JF (2007). Endoplasmic reticulum stress and the unfolded protein response regulate genomic cystic fibrosis transmembrane conductance regulator expression.. Am J Physiol Cell Physiol.

[51] Bodas M, Min T, Mazur S, Vij N (2010). Critical modifier role of membrane-cystic fibrosis transmembrane conductance regulator-dependent ceramide signaling in lung injury and emphysema.. J Immunol.

[52] Hogg JC, Chu FS, Tan WC, Sin DD, Patel SA (2007). Survival after lung volume reduction in chronic obstructive pulmonary disease: insights from small airway pathology.. American journal of respiratory and critical care medicine.

[53] Zheng JP, Kang J, Huang SG, Chen P, Yao WZ (2008). Effect of carbocisteine on acute exacerbation of chronic obstructive pulmonary disease (PEACE Study): a randomised placebo-controlled study.. Lancet.

[54] Decramer M, Rutten-van Molken M, Dekhuijzen PN, Troosters T, van Herwaarden C (2005). Effects of N-acetylcysteine on outcomes in chronic obstructive pulmonary disease (Bronchitis Randomized on NAC Cost-Utility Study, BRONCUS): a randomised placebo-controlled trial.. Lancet.

[55] Fahy JV, Dickey BF (2010). Airway mucus function and dysfunction.. N Engl J Med.

