# Supplementary figures and images for: A Pharmacologic Approach to Acquired Cystic Fibrosis Transmembrane Conductance Regulator Dysfunction in Smoking Related Lung Disease
Source: PLoS One. 2012 Jun 29;7(6):e39809. doi: 10.1371/journal.pone.0039809 (PMC3387224; doi:10.1371/journal.pone.0039809)

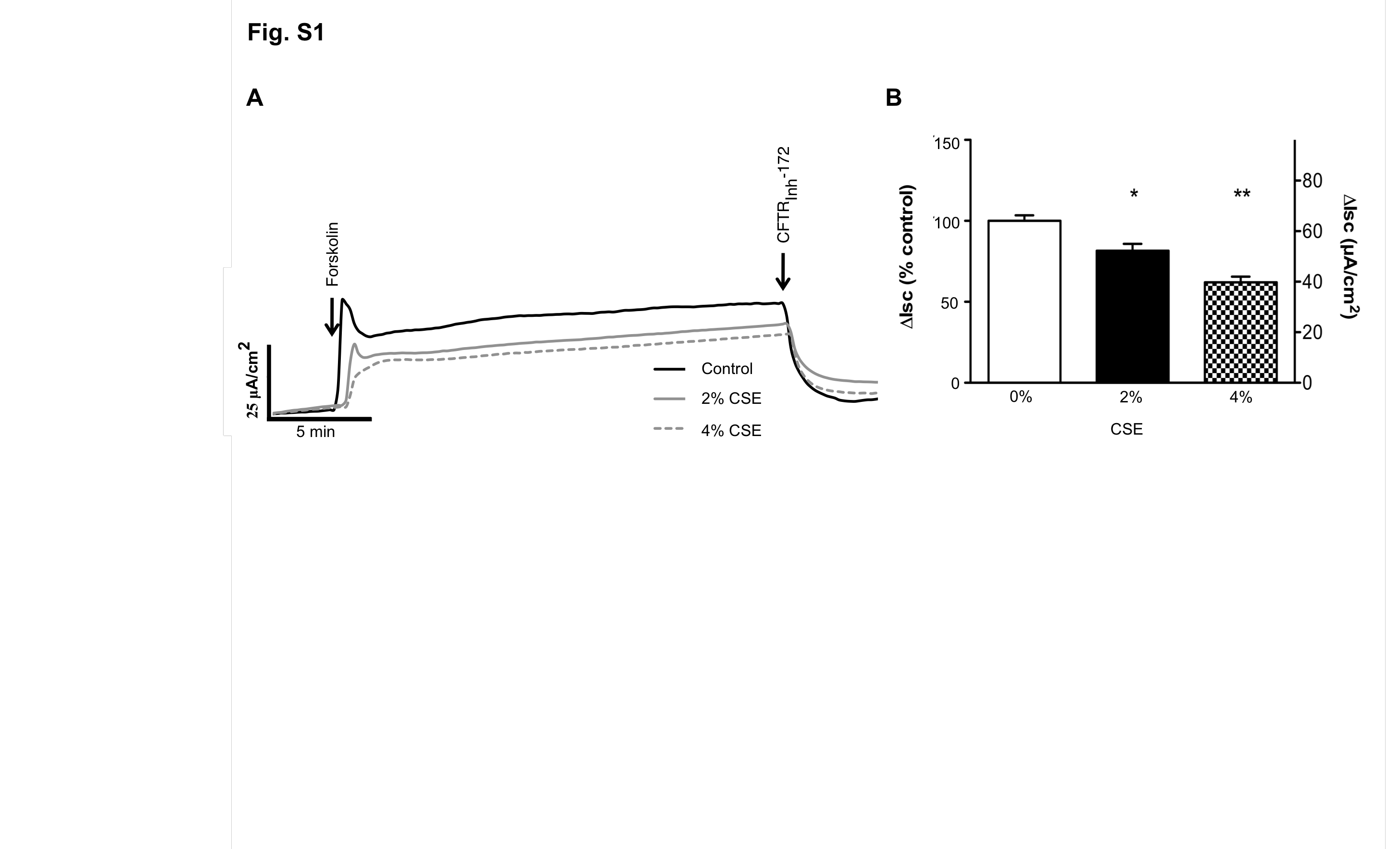

Supplement: Figure S1 — (A) Representative Ussing chamber tracings from Calu-3 cells grown at air-liquid interface, then exposed to CSE (2%) for 24 h and studied under voltage clamp conditions as shown in Fig. 1A. (B) Summary data of experiments shown in (A). ΔIsc is shown following stimulation with forskolin (20 µM) *P<0.05, n = 8 **P<0.01, n = 8. (TIFF) [file pone.0039809.s001.tif]

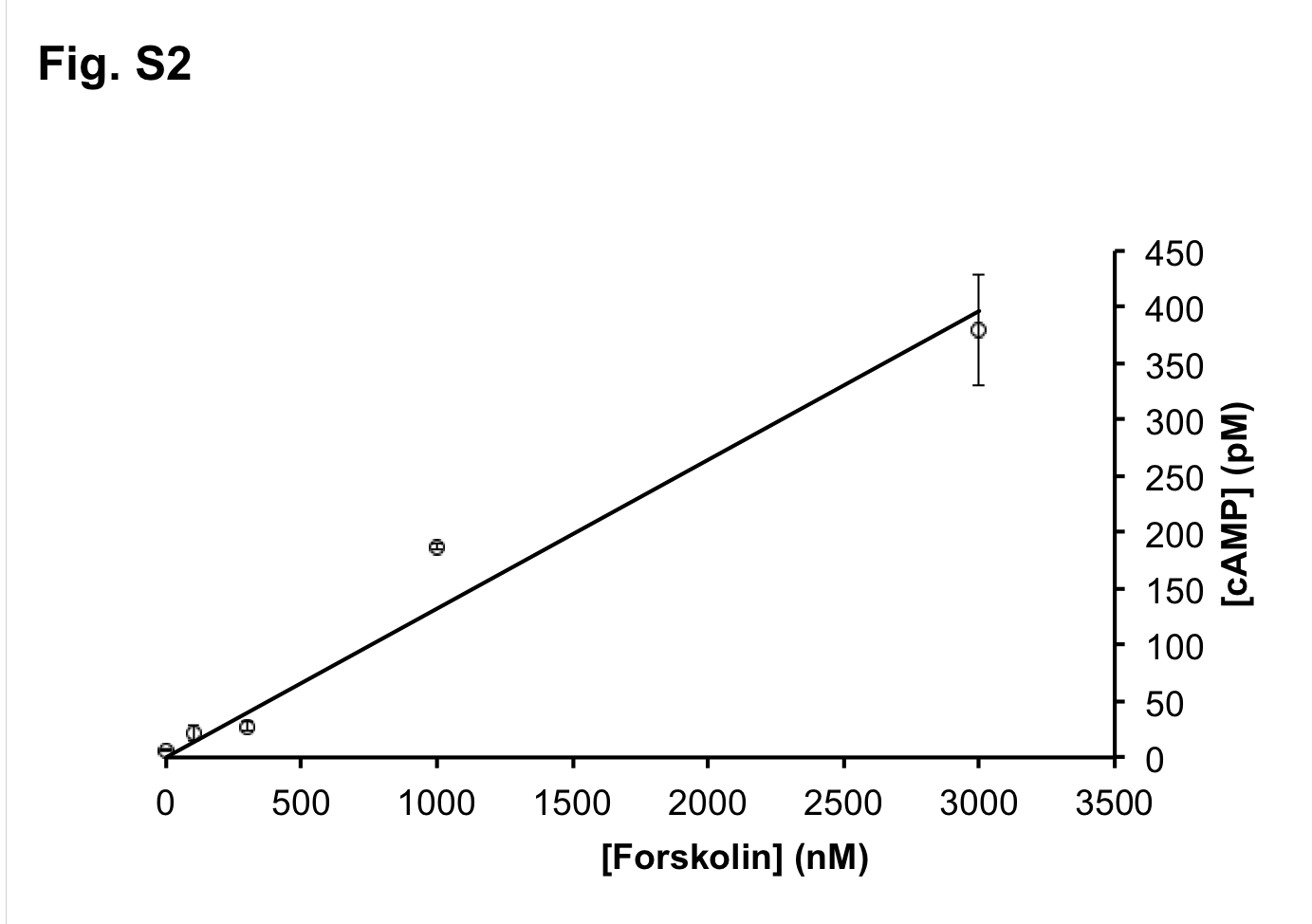

Supplement: Figure S2 — Cellular cAMP levels determined by colorimetric assay in non-CF HBE monolayers stimulated with forskolin (0, 100, 300 and 1000 nM) for 10 min prior to lysis, n = 3/condition. (TIFF) [file pone.0039809.s002.tif]

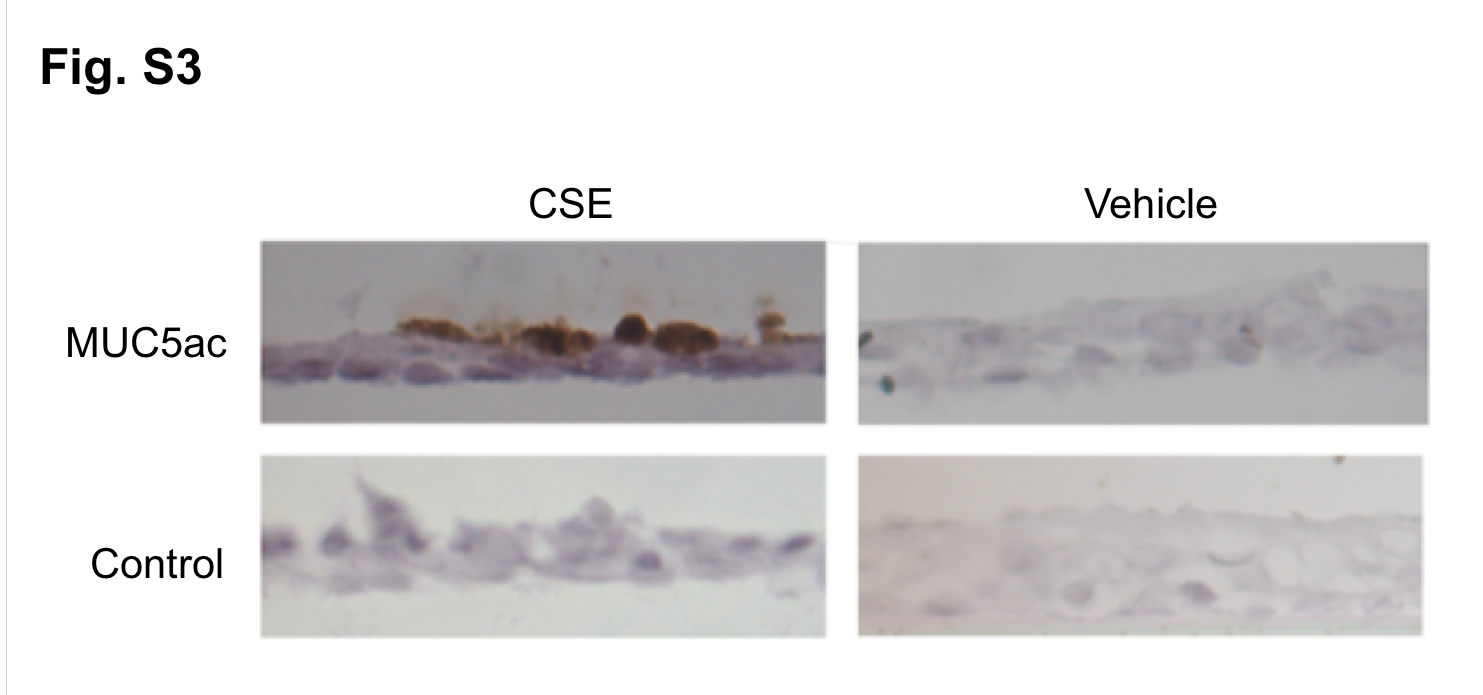

Supplement: Figure S3 — MUC5ac expression is altered by CSE exposure. MUC5ac staining is shown from representative sections of HBE cells exposed to CSE (2%) or vehicle control for 24 h. Control slides were stained in the absence of primary anti-MUC5ac antibody. (TIFF) [file pone.0039809.s003.tif]

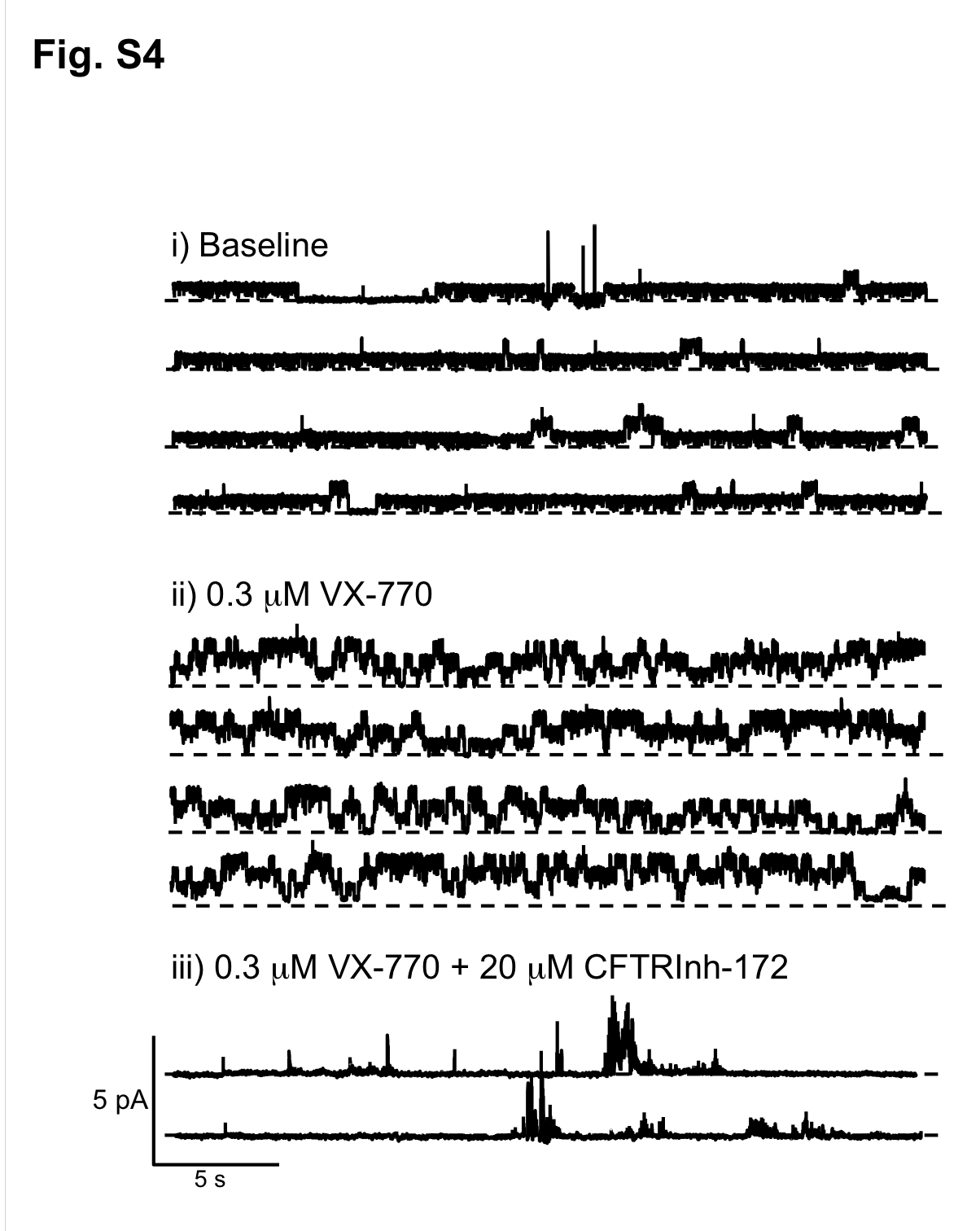

Supplement: Figure S4 — Effect of ivacaftor on open channel probability. Inside-out membrane patches were obtained from NIH3T3 cells transduced with WT CFTR and single channel conductance tracings recorded with control (i, Po = 0.4±0.04); 0.3 µM ivacaftor (ivacaftor; ii, Po = 0.8±0.04); and 0.3 µM ivacaftor +20 µM CFTRInh-172 (iii). All recordings performed in the presence of 75 nM PKA +1 mM ATP. Dotted line represents closed channel configuration. Summary data of these experiments was shown previously by Van Goor et al. [17]. (TIFF) [file pone.0039809.s004.tif]

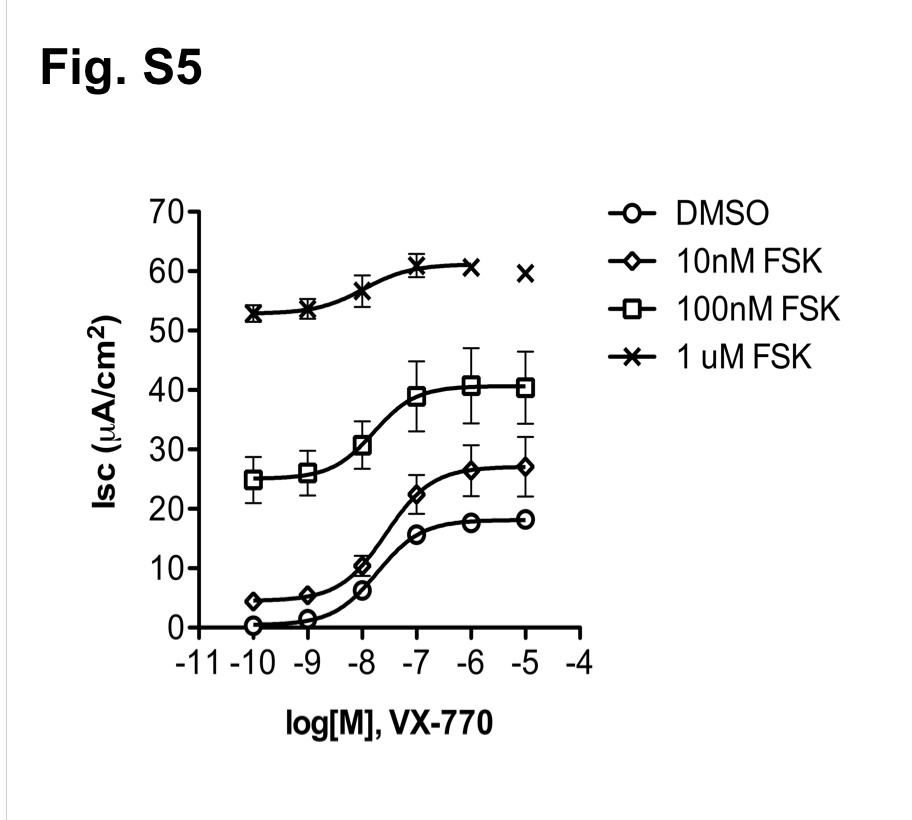

Supplement: Figure S5 — Effect of forskolin prestimulation on ivacaftor induced Isc. Prior to ivacaftor addition, varying concentrations of forskolin were administered as shown to non-CF HBE cells, then Isc plotted following sequential addition of increasing concentrations of ivacaftor (ivacaftor), n = 3/condition. (TIFF) [file pone.0039809.s005.tif]

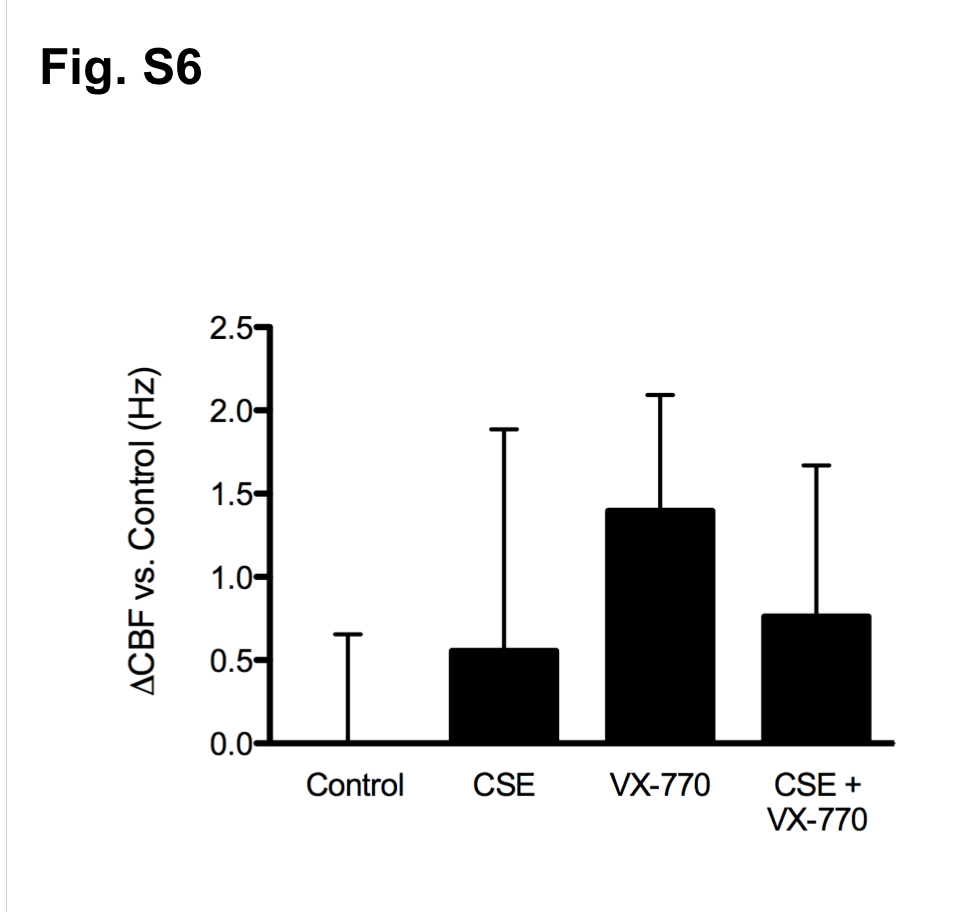

Supplement: Figure S6 — Effect of CSE exposure on ciliary beating. CSE (2%, apical), ivacaftor (ivacaftor; 10 µM, basolateral), both agents, or vehicle control was applied to primary human bronchial epithelial monolayers for 24 hrs, and then the change in ciliary beat frequency (CBF) from pretreatment baseline was assessed by Hoffman contrast microscopy at 4–5 ROI for each well. N = 3/condition. (TIFF) [file pone.0039809.s006.tif]
